# Evaluating China's primary healthcare services' efficiency and spatial correlation: a three-stage DEA-Malmquist model

**DOI:** 10.3389/fpubh.2024.1366327

**Published:** 2024-06-19

**Authors:** Rui Huang, Wan Li, Baoguo Shi, Hao Su, Jing Hao, Chuanjun Zhao, Juhong Chai

**Affiliations:** ^1^Department of Management, School of Management, Minzu University of China, Beijing, China; ^2^Department of Economics, School of Economics, Minzu University of China, Beijing, China; ^3^Department of National Security, School of National Security, Minzu University of China, Beijing, China

**Keywords:** primary healthcare, service efficiency, three-stage DEA-Malmquist model, spatial autocorrelation, China

## Abstract

**Introduction:**

Enhancing the efficiency of primary healthcare services is essential for a populous and developing nation like China. This study offers a systematic analysis of the efficiency and spatial distribution of primary healthcare services in China. It elucidates the fundamental landscape and regional variances in efficiency, thereby furnishing a scientific foundation for enhancing service efficiency and fostering coordinated regional development.

**Methods:**

Employs a three-stage DEA-Malmquist model to assess the efficiency of primary healthcare services across 31 provincial units in mainland China from 2012 to 2020. Additionally, it examines the spatial correlation of efficiency distribution using the Moran Index.

**Results:**

The efficiency of primary healthcare services in China is generally suboptimal with a noticeable declining trend, highlighting significant potential for improvement in both pure technical efficiency and scale efficiency. There is a pronounced efficiency gap among provinces, yet a positive spatial correlation is evident. Regionally, efficiency ranks in the order of East > Central > West. Factors such as GDP per capita and population density positively influence efficiency enhancements, while urbanization levels and government health expenditures appear to have a detrimental impact.

**Discussion:**

The application of the three-stage DEA-Malmquist model and the Moran Index not only expands the methodological framework for researching primary healthcare service efficiency but also provides scientifically valuable insights for enhancing the efficiency of primary healthcare services in China and other developing nations.

## 1 Introduction

In the current context of global health governance, the efficiency and effectiveness of the primary healthcare delivery system have become critical for countries in their pursuit of health coverage, improving population health and preventing and controlling infectious diseases (1–3). As the cornerstone of integrated health services (4), the primary healthcare delivery system not only covers a wide range of healthcare services across the life cycle, from disease prevention to treatment and management of chronic diseases (5, 6), but also plays a critical role in improving population health outcomes, reducing the need for resource-intensive secondary healthcare services and increasing economic output (7). However, despite the fact that PHC systems are widely established and promoted globally, significant challenges remain regarding the evaluation and optimization of their efficiency.

In China, despite substantial increases in government investment in the PHC system since the 2009 healthcare reform—with funding for PHC services rising from 843.198 billion yuan in 2012 to 219.419 billion yuan in 2020—and ongoing growth in the number of institutions and personnel, efficiency issues continue to hinder the enhancement of basic healthcare service provision and quality (8). Given the challenges posed by an aging population and the increasing prevalence of chronic diseases, improving the efficiency of the PHC system and optimizing resource allocation have become critical concerns.

The COVID-19 pandemic has underscored the critical need to enhance the efficiency of primary healthcare services. As the first line of defense in disease prevention and control, the efficiency of these services is directly linked to effective epidemic management and public health safety. Throughout the pandemic, primary healthcare facilities faced numerous challenges, including a surge in patient numbers, resource limitations, and service disparities. Research has demonstrated that optimizing resource allocation, strengthening human resource management, implementing digital healthcare solutions (9–11), and boosting community engagement can substantially improve service efficiency and alleviate epidemic-related pressures. Nonetheless, technical, policy, and sociocultural hurdles continue to impede efficiency enhancements. By bolstering the efficiency and resilience of primary healthcare systems, we can forge a more robust public health infrastructure capable of confronting future health crises.

In recent years, there has been a growing academic interest in the efficiency of PHC services, with numerous studies employing DEA techniques to evaluate this area. These studies have provided crucial insights into potential avenues for enhancing PHC efficiency. However, they often focus narrowly on localized, single-efficiency metrics, overlooking the significant impacts of external environmental factors and internal management efficiency on PHC service effectiveness (12–16). Previous research highlights the importance of considering both external influences and other contributing factors in efficiency assessments (17). Moreover, studies exploring the spatial distribution and regional disparities in PHC efficiency remain limited. For a vast and populous developing country such as China, this gap in research restricts a comprehensive understanding of the underlying causes behind variations in primary healthcare service efficiency and impedes the formulation of targeted strategies.

To address the limitations of existing studies, this study proposes the use of a three-stage DEA-Malmquist model to systematically assess the efficiency of PHC systems in China. Unlike traditional DEA models, the three-stage DEA-Malmquist model effectively isolates and assesses the impacts of external environmental factors (18), stochastic perturbations, and managerial efficiencies on service efficiency (19). This methodology, which has proven effective in other fields, provides a more accurate and comprehensive assessment of efficiency at the grassroot level in China. Additionally, this study analyzes the spatial distribution patterns of PHC efficiency across 31 provincial units in China and explores the spatial autocorrelation among these efficiencies using Moran's I index. Despite the vast regional differences previously documented, the questions of what kind of spatial pattern exists and whether there is any spatial correlation remain open. This innovative approach not only reveals the geographical differences in the efficiency of PHC services in China but also provides a scientific basis for formulating strategies for regional coordinated development.

The remaining sections of the study are structured in the following manner. Section 2 is an introduction to the theoretical background. Section 3 provides a comprehensive overview of the research on the efficiency of PHC and studies on spatial autocorrelation. Section 4 presents the three-stage DEA-Malmquist model and its associated variables. Section 5 examines the general effectiveness of PHC organizations in China, the variation in effectiveness among 31 provincial units, the factors in the environment that impact effectiveness, the spatial pattern of effectiveness evaluated using Moran's I index, and robustness tests were performed. Section 6 presents a comprehensive analysis and evaluation, while Section 7 offers definitive summary and final remarks.

## 2 Theoretical background

Efficiency is a multi-faceted concept extensively explored across various fields, particularly emphasizing the optimal relationship between inputs and outputs (20). In economics, efficiency primarily concerns the ideal utilization of resources to maximize production or meet the greatest possible demand. Within health economics, the focus shifts to achieving the maximum healthcare service output with minimal resource input (21). When applied to the management and delivery of primary healthcare services, efficiency centers on how to deliver the highest quality of care using the least resources (22), thereby addressing broader healthcare needs more effectively. This approach is crucial in optimizing healthcare delivery systems, ensuring that resources are used most effectively to improve patient outcomes and service quality.

Primary healthcare services, grounded in public goods theory, are defined by their non-exclusivity and non-rivalry. Non-exclusivity ensures that once services are available, they are accessible to everyone, regardless of whether they have paid, as observed in disease prevention programs that benefit public health broadly. Non-rivalry means that services provided to one person do not reduce their availability to others, which is exemplified by community health education that does not diminish in quality as more participants engage (23). This positions primary healthcare as a public good, requiring collective funding and action due to its nature, which often leads to market failures, where the private sector cannot adequately provide these essential services.

The public goods theory underscores the essential nature of primary healthcare services as fundamental rights, emphasizing the need for government intervention to prevent underprovision and ensure universal access (24). This theory posits that strategic public policies and financial support are crucial to addressing market failures and achieving efficient provision of primary healthcare services (25). Efficiency issues within primary healthcare are complex, involving the misallocation of resources, inefficiencies in service delivery, and gaps in accessibility and quality. To tackle these challenges, comprehensive strategies are necessary, which include adopting innovative technologies, reforming management practices, and optimizing resource allocation. These measures are aimed at enhancing service delivery and improving patient outcomes, ultimately supporting the broader goals of health equity and effectiveness.

Applying public goods theory to primary healthcare services, this study builds upon the foundational concepts of non-exclusivity and non-competitiveness from the existing literature (26) while also exploring the government's role through a systematic analysis that deepens our understanding of healthcare policies and practices. Utilizing a three-stage DEA model, this research meticulously assesses the impact of environmental variables and random errors on efficiency, introducing a novel methodological approach to evaluating primary healthcare service efficiency. By integrating these methods and theoretical frameworks, the study offers empirical support and strategic insights for effectively utilizing public resources, enhancing service quality, and achieving equity in primary healthcare. These findings aim to assist in developing and refining public health policies to better fulfill the health needs of the public.

## 3 Literature review

### 3.1 PHC efficiency

The effectiveness of PHC services has consistently been a prominent subject in international studies. Contemporary academic research focuses on the operational efficiency of PHC organizations and the allocation efficiency of PHC resources. Some studies also examine the comparative efficiency of PHC organizations in urban and rural areas. Paul conducted a two-stage DEA analysis to assess the efficiency of rural PHC services in Burkina Faso (27). The results indicated that the efficiency was poor, and that distance had a crucial role in influencing the efficiency of PHC organizations. A study conducted on direct healthcare services in rural Greece, utilizing a limited model, revealed that the primary cause of inefficiency in PHC services is mostly attributed to low levels of technical efficiency (28). Farhad et al. employed the DEA approach and regression modeling to assess the efficiency of several basic healthcare facilities in Afghanistan. Their findings indicate that as the level of the institution increases, its efficiency also increases (29).

The efficiency of primary healthcare services in China has attracted considerable academic interest in recent years, particularly in the context of the country's deepening and advancing healthcare reforms. Cheng et al. have undertaken comparative analyses on the efficacy of PHC institutions from an urban–rural standpoint (30). Zhou et al. utilized the DEA-Malmquist index to examine the efficiency of urban and rural PHC organizations in China (31). A study by Zhao et al. found a decline in the efficiency of primary healthcare services, and the main reason for this was a slowdown in technological change (32). We have organized these previous related studies, as shown in Table 1.

**Table 1:** Overview of previous studies on the efficiency of primary healthcare services.

**References**	**Research method**	**Main findings**
Marschall and Flessa (27)	Two-stage DEA	Efficiency in rural primary health care is low; distance impacts efficiency.
Oikonomou et al. (28)	Restricted DEA	Low technical efficiency is the main reason for poor overall efficiency.
Cheng et al. (30)	Bootstrap-DEA	Higher efficiency in rural health centers compared to urban health service centers.
Zhong et al. (33)	DEA-Malmquist	Primary healthcare services show low efficiency.
Farewar et al. (29)	DEA and regression models	Higher institutional levels correlate with higher efficiency in primary health care facilities.
Yan et al. (16)	DEA-Malmquist	Significant potential for improvement in primary health care efficiency; regional disparities exist.
Chen et al. (34)	DEA-Malmquist	Good efficiency in primary health care services, with room for improvement.
Zhou et al. (31)	DEA-Malmquist	Urban primary healthcare services are more efficient than rural ones.
Zhao et al. (32)	super-SBM model, Malmquist index	Decline in primary healthcare efficiency, mainly due to a slowdown in technological innovation.

In reviewing the existing literature (16, 33), it becomes apparent that there is a general consensus within the academic community regarding the issue of suboptimal efficiency in primary healthcare services (8, 35, 36). Nevertheless, due to regional disparities in socioeconomic development and healthcare infrastructure, research findings vary, indicating the need for further investigation (31, 34, 37). Future studies should consider diverse social contexts (38) to better understand and address these inconsistencies in healthcare service efficiency.

In terms of research methodology, Data Envelopment Analysis (DEA) has been a pivotal tool for assessing healthcare service efficiency since the 1980s. Nunamaker first introduced the DEA methodology to the field of medicine and healthcare in 1983 (39). Since then, DEA has been extensively applied across various sectors, including hospitals and primary healthcare. Despite the prevalence of traditional DEA approaches in analyzing primary healthcare service efficiency, recent years have seen significant advancements in DEA applications (30). Innovative models such as the Slacks-Based Measure (SBM-DEA), super-efficiency DEA, Network DEA Model (40), and multi-phase DEA models, including two-phase (41), three-phase, and four-phase (18, 42, 43), have been increasingly adopted. These enhancements have proven to offer more scientific and reliable efficiency measurements by accounting for a broader range of factors affecting efficiency. The ongoing refinement of these evaluation methods provides a more robust tool for conducting research in this field, enhancing the empirical rigor and applicability of the findings.

### 3.2 Spatial autocorrelation

Sun and Yao (44) study, titled “Spatial Analysis,” marked the first instance of quantifying geography. This groundbreaking work laid the foundation for the emergence of spatial econometrics. With the increasing use of spatial econometric methods in the health field, researchers have started applying spatial analysis techniques to assess the availability of health services and the geographical distribution of medical resources. This is done to address the limitations of traditional evaluation methods, which do not consider spatial location information. A study conducted on the allocation of health resources in the Brazilian Amazon revealed the presence of a positive spatial autocorrelation in their distribution (45). Shi et al. conducted a study to assess the spatial distribution of top-tier hospitals in China (46). Their findings revealed that these hospitals were not evenly dispersed and had notable variations in spatial patterns. Guo et al. employed a spatial gravity model to assess the spatial correlation network of China's concentration capacity of health resources and the factors that influence it (47). Xiong et al. conducted a study on the spatial analysis of PHC in Hong Kong, focusing on its accessibility and availability (48).

Moran's I index is the predominant research tool utilized in spatial correlation research for efficiently analyzing spatial autocorrelation. Zhu et al. analyzed the effectiveness of healthcare resource allocation in China. They utilized Moran's index to measure spatial autocorrelation and discovered a positive correlation in efficiency. Furthermore, they observed that this correlation is steadily growing over time (49). Liu and Wang conducted a study on the spatial autocorrelation and clustering of the effectiveness of basic public health services in rural areas across different provinces in China. They utilized Moran's index to analyze the data and found strong evidence of a positive spatial correlation (50). Sun et al. conducted a study on the regional heterogeneity and influencing factors of health expenditure efficiency in Chinese provincial units. They discovered a notable spatial clustering phenomenon in the health expenditure efficiency of these units (51). Yu et al. assessed the spatial correlation of efficiency by employing the global Moran's I index and local Moran's I index to analyze the efficiency of healthcare expenditure in Western China. The study confirmed a positive connection in the distribution of efficiency (52).

The existing research on primary care efficiency has made significant advancements in both theoretical understanding and methodological approaches, providing a solid basis for our study. Nevertheless, the present research still has certain limitations, and it is necessary to further enhance the evaluation techniques for efficiency. Additionally, there is an urgent need to address the spatial distribution pattern of efficiency and other related concerns. This study utilized a three-stage DEA-Malmquist model to systematically analyze the efficiency of PHC systems in 31 provincial units in mainland China. The analysis was based on a comprehensive review and summary of previous relevant literature. The study used the latest official publicly released data from 2012 to 2020. The spatial distribution of efficiency among provincial units in China is depicted using Moran's I index. This index serves as a scientific reference for the development of PHC systems in China and other similar developing populous countries.

## 4 Methodology

### 4.1 Three-stage DEA-Malmquist model

DEA is a method based on the multiple-input multiple-output technique, predominantly utilized to calculate efficiency. This model excels in evaluating the operational efficiency of decision-making units handling multiple inputs and outputs (53, 54) an aspect crucial for analyzing efficiency in healthcare services. A distinct advantage of the DEA model lies in its ability to assess the relationship between multiple resource inputs and service outputs without requiring preset weights (55, 56). This feature has been extensively validated and supported in the literature (57), reinforcing its applicability and robustness in efficiency studies within the healthcare sector.

The three-stage DEA model utilized in this study builds on traditional DEA models by specifically accounting for the impact of environmental factors and random errors on efficiency evaluations. This enhancement aligns with the findings (58) by Yang et al. and supports the assertion by Hu et al. regarding the model's efficacy in pinpointing and enhancing the operational efficiency of decision-making units (DMUs) (59). A significant advantage of the three-stage DEA model is its lack of priori assumptions about data distribution forms, which enhances its flexibility in handling complex datasets. Moreover, the second stage of the three-stage DEA model incorporates a Stochastic Frontier Analysis (SFA), effectively distinguishing random noise from true efficiency measurements. This separation ensures the precision and scientific validity of the results. The SFA method evaluates the efficiency performance of DMUs under the influence of uncontrollable environmental factors, a technique that is both widely used and recognized in the literature (60, 61). The parametric nature of SFA allows for the exploration of specific functional forms of DMUs, enriching the analysis beyond the non-parametric efficiency scores provided by traditional DEA. This layered approach significantly deepens the study's insights into service efficiency.

The steps for the three-stage DEA model calculation are as follows. First, the analysis of the conventional DEA model is carried out in the first stage. The first stage carries out the analysis of the traditional DEA model. The BCC model with variable returns to scale in the DEA approach is used to decompose the technical efficiency (TE) into the product of pure technical efficiency (PTE) and scale efficiency (SE) to solve the problem of the effectiveness of the decision unit with variable returns to scale (62). That is, TE = PTE ^*^ SE, where pure technical efficiency PTE denotes the managerial and technical level of the decision-making unit, and scale efficiency SE denotes the scale of the decision-making unit's input of resources (63). This study uses the input-oriented BCC model. The DEA analysis in the first phase did not consider the effects of environmental factors and random disturbances. Therefore, in the second stage, we need to decompose these influences, i.e., the slack variables computed in the first stage, to ensure that all the decision units are in the same external environment (19). The following SFA regression function was constructed:


$$\begin{array}{l} S_{ni}=f\left(Z_{i};\beta_{n}\right)+\nu_{ni}+\mu_{ni};i=1,2,\cdots \;,I;n=1,2,\cdots \;,N \end{array}$$

where *S*_*ni*_ is the slack value of the *n* input of the *i* decision unit; *Z*_*i*_ is the environmental variable, and β_*n*_ is the coefficient of the ecological variable; ν_*ni*_+μ_*ni*_ is the mixed error term, in which ν_*ni*_ denotes random disturbances and μ_*ni*_ indicates managerial inefficiency. $\nu \tilde{\;}N\left(0,{\sigma_{v}}^{2}\right)$ is a random error term that represents the effect of random disturbances on the input slack variables. μ is administrative inefficiency, which means the effect of organizational factors on the input slack variable, assumed to follow a normal distribution truncated at the null point, $\mu \tilde{\;}N^{+}\left(0,{\sigma_{\mu }}^{2}\right)$.

Using the results estimated from the SFA regression model above, the inputs to the decision unit are adjusted using the following formula:


$$\begin{array}{l} X_{ni}^{A}=X_{ni}+\left[\max\left(f\left(Z_{i};{\overset{\wedge }{\beta }}_{n}\right)\right)-f\left(Z_{i};{\overset{\wedge }{\beta }}_{n}\right)\right]+\left[\max\left(\nu_{ni}\right)-\nu_{ni}\right]\;\\ \;i=1,2,\cdots \;,I;n=1,2,\cdots \;,N \end{array}$$

$X_{ni}^{A}$ is adjusted inputs; *X*_*ni*_ is pre-adjusted inputs; $\left[\max\left(f\left(Z_{i};{\overset{\wedge }{\beta }}_{n}\right)\right)-f\left(Z_{i};{\overset{\wedge }{\beta }}_{n}\right)\right]$ indicates adjustments to external environmental factors; [max(ν_*ni*_)−ν_*ni*_] is designed to eliminate the effects of the external environment on efficiency by placing all decision-making units in the same external environment.

The process of DEA analysis is repeated in the third stage. However, the adjusted input variables are substituted into the model instead of the initial input variables, and the output variables remain the initial output variables. At this point, the variables have been removed from the influence of external environmental factors, and therefore, a relatively accurate efficiency assessment is obtained.

Because the three-stage DEA analysis can only obtain static efficiency analysis results, the Malmquist index is used to analyze the dynamic changes in efficiency [25]. The Malmquist index model is formulated as follows:


$$\begin{array}{l} \;M\left(x_{i}^{t+1},y_{i}^{t+1},x_{i}^{t},y_{i}^{t}\right)=TFP\\ \;=\sqrt{\frac{D_{i}^{t+1}\left(x^{t},y^{t}\right)}{D_{i}^{t+1}\left(x^{t+1},y^{t+1}\right)}\times \frac{D_{i}^{t}\left(x^{t},y^{t}\right)}{D_{i}^{t}\left(x^{t+1},y^{t+1}\right)}}\\ =\frac{D_{i}^{t+1}\left(x^{t},y^{t}\right)}{D_{i}^{t+1}\left(x^{t+1},y^{t+1}\right)}\sqrt{\frac{D_{i}^{t+1}\left(x^{t},y^{t}\right)}{D_{i}^{t}\left(x^{t+1},y^{t+1}\right)}\times \frac{D_{i}^{t+1}\left(x^{t},y^{t}\right)}{D_{i}^{t}\left(x^{t+1},y^{t+1}\right)}} \end{array}$$

$$\begin{array}{l} \;=EFFCH\times TECH\\ =\left(PECH\times SECH\right)\times TECH \end{array}$$

TFP>1 means that the current production efficiency is higher than in the previous period, TFP = 1 means that the current production efficiency is the same as in the last period, and TFP <1 means that the current production efficiency is lower than in the previous period. Total factor productivity (TFP) can be decomposed into technical efficiency change (EFFCH) and technological progress (TECH), which, in turn, can be decomposed into pure technical efficiency change (PECH) and scale technical efficiency change (SECH).

### 4.2 Spatial autocorrelation

Spatial autocorrelation is a metric used to quantify the level of spatial clustering of variables within a given area. It is divided into two primary categories: global spatial autocorrelation and local spatial autocorrelation. Moran's index is commonly employed to assess spatial autocorrelation. Moran's index is a widely used statistic for measuring the correlation between neighboring regions in a spatial context. The global Moran's index is utilized to assess the spatial correlation dynamics of the PHC system over the entire study area, employing the following formula:


$$I=\frac{\sum_{i=1}^{n}\sum_{j=1}^{n}w_{ij}(X_{i}{-}^{\bar{X}})(X_{j}{-}^{\bar{X}})}{S^{2}\sum_{i=1}^{n}\sum_{j=1}^{n}w_{ij}}$$

Xi and Xj are the efficiency of PHC services in cities i and j, respectively; S^2^ is the variance of the efficiency, and X is the mean of the efficiency. The global Moran's I index *I* is between [−1,1]; when *I* expects a value of 0, it indicates a positive spatial correlation, and the close it is to 1, the more intimate the relationship between spatial units is. When *I* expects a value of 0, it means a negative spatial correlation, and the closer it is to −1, the more significant the gap between neighboring cells in space is. When *I* = 0, it indicates a spatially random distribution. The localized Moran's I index is used to measure the spatial aggregation of provinces and cities with localized neighboring towns and areas. The localized agglomeration characteristics of PHC service efficiency in each province and city are classified into four types: high–high agglomeration, high–low agglomeration, low–high aggregation, and low–low agglomeration. The calculation formula is as follows:


$$I_{i}=\frac{\left(X_{i}{-}^{\bar{X}}\right)}{S^{2}}\sum_{j=1}^{n}W_{ij}(X_{j}{-}^{\bar{X}})$$

### 4.3 Data and methods

#### 4.3.1 Input–output variables

The selection of the indicator system is the core of DEA efficiency evaluation and has a critical impact on the assessment results. The input indicators used in previous relevant studies were mainly human and financial, as the number of health personnel, medical expenditures, and beds and the output indicators included the number of consultations, hospitalizations, discharges, and bed occupancy rate (64). Considering the differences in the caliber of assessment of PHC system in various countries, we sort out the input–output indicators used in studies related to assessing the efficiency of PHC services in China (Table 2).

**Table 2:** Selection of input–output variables in previous studies.

**References**	**Input variables**	**Output variables**	**Method**
Cheng et al. (30)	Health personnel, Number of beds	Outpatient visits, inpatient bed-days	Bootstrap-DEA
Zhong et al. (33)	Health technical staff, Number of beds, Number of equipment	Outpatient and emergency visits, discharges	DEA-Malmquist
Yan et al. (16)	Number of beds, Health technical staff, Number of institutions	Outpatient visits, inpatient visits	DEA-Malmquist
Chen et al. (34)	Number of institutions, Number of beds, Practicing physicians, Registered nurses	Outpatient visits, Inpatient visits, Home health service visits	DEA-Malmquist
Zhou et al. (31)	Number of institutions, Number of beds, Health technical staff	Outpatient and emergency visits, Discharges	DEA-Malmquist
Zhao et al. (32)	Number of institutions, Number of beds, Health technical staff	Outpatient visits, inpatient visits	Super-SBM Model, Malmquist index

Synthesizing the above research (32, 33) and following the principles of representativeness, stability, and independence in the selection of indicators (65, 66) and taking into account the characteristics of the PHC system and the availability of data, this study ultimately selected the number of PHC institutions, health personnel, and beds as the input indicators and the numbers of consultations and admissions as the output indicators, as shown in Table 3.

**Table 3:** PHC input–output indicators and environmental variables.

**Type**	**Name**	**Definition**	**Unit**
Input indicators	Number of health institutions	Number of PHC institutions	Unit
	Number of health personnel	Health technicians, rural doctors and health workers, other technicians, etc.	Person
	Number of beds	Number of fixed real beds in PHC organizations at the end of the year	Bed
Output indicators	Number of consultations	Total attendance numbers for all consultations in PHC facilities	Ten Thousand
	Number of hospital admissions	Number of hospitalizations	Ten Thousand
Environmental variables	GDP per capital	GDP per capita	Yuan
	Population density	Number of people per unit of land area	Per Sq. Kilo
	Urbanization rate	Proportion of urban resident population to total resident population	%
	Government health expenditure	Government funds invested in health services, Medicare subsidies, and other endeavors	Billions

Source: National Health Commission (67).

#### 4.3.2 Environmental variables

Environmental variables should be selected in such a way that they have an impact on the subject of the study but are not subjectively moderated (66). In terms of their selection, previous related studies have mostly looked at economic development level, urbanization level, human resources for health, population density, education level, and government health expenditures (65). This study selects four environmental variables: per capita GDP, population density, urbanization rate, and government health expenditures (68). The following system of evaluation indicators was ultimately constructed (Table 3).

#### 4.3.3 Data sources

The data in this study cover the relevant indicators of PHC organizations in 31 provinces in China from 2012 to 2020, which are from the China Health and Family Planning Statistical Yearbook, China Health and Healthcare Statistical Yearbook, and China Statistical Yearbook, and the descriptive statistics of the indicator data are shown in Table 3. To facilitate regional comparisons, according to the division criteria of the National Bureau of Statistics, the 31 provinces are categorized into three regions: east, central, and west. The east includes the 11 provinces, namely, Beijing, Tianjin, Hebei, Liaoning, Shanghai, Jiangsu, Zhejiang, Fujian, Shandong, Guangdong, and Hainan; the central region includes the 8 provinces, namely, Shanxi, Jilin, Heilongjiang, Anhui, Jiangxi, Henan, Hubei, and Hunan; and the western region comprises the 12 provinces, namely, Inner Mongolia, Guangxi, Chongqing, Sichuan, Guizhou, Yunnan, Tibet, Shaanxi, Gansu, Qinghai, Ningxia, and Xinjiang.

## 5 Result

### 5.1 Static efficiency analysis

#### 5.1.1 Stage I: traditional DEA analysis

**Figure 1:**
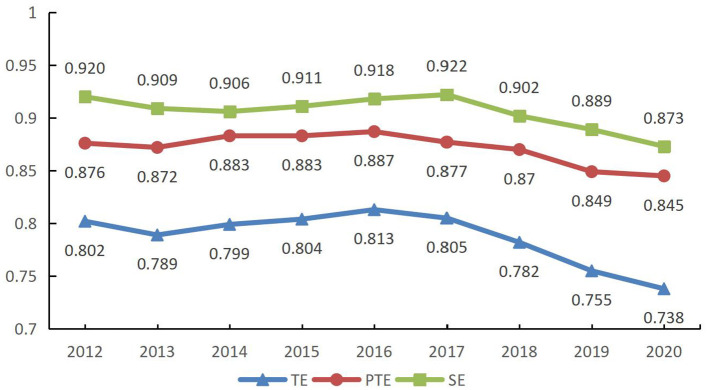
Efficiency values for the first phase (2012–2020).

From 2012 to 2020, the efficiency of China's PHC system showed a floating downward trend, with a low efficiency mean (Figure 1, Table 4), while both pure technical efficiency and scale efficiency were essential constraints on overall efficiency. In the study comparing the efficiency values of the first and third phases, there was an overall decrease in the mean value of efficiency after removing the effects of random disturbances and environmental factors. The mean value of technical efficiency decreased from 0.802 to 0.747, the mean value of pure technical efficiency decreased from 0.876 to 0.871, and the mean value of scale efficiency decreased from 0.92 to 0.847. This indicates that the previous traditional DEA method failed to reflect the true efficiency level of the PHC system because it did not exclude the influence of random interference and environmental factors, and the efficiency value was overestimated.

**Table 4:** PHC efficiency in 31 provincial units.

**Province**	**Stage 1**					**Stage 3**					
	**TE**	**PTE**	**SE**	**RTS**	**Rank**	**TE**	**PTE**	**SE**	**RTS**	**Rank**	**Compare**
Beijing	1	1	1	-	1	0.644	0.889	0.724	irs	20	↑
Tianjin	0.887	1	0.887	irs	13	0.731	1	0.731	irs	18	↑
Hebei	0.848	0.853	0.995	drs	16	0.833	0.846	0.985	irs	15	↑
Shanxi	0.451	0.462	0.975	irs	30	0.437	0.509	0.859	irs	30	↑
Inner Mongolia	0.51	0.536	0.951	irs	27	0.477	0.596	0.801	irs	27	↑
Liaoning	0.595	0.603	0.985	irs	25	0.618	0.694	0.89	irs	21	↓
Jilin	0.488	0.523	0.933	irs	29	0.471	0.598	0.789	irs	28	↑
Heilongjiang	0.506	0.542	0.933	irs	28	0.544	0.687	0.792	irs	24	↓
Shanghai	1	1	1	-	2	0.806	1	0.806	irs	16	↑
Jiangsu	0.887	1	0.887	drs	14	0.911	0.924	0.986	irs	11	↓
Zhejiang	1	1	1	-	3	1	1	1	-	1	-
Anhui	0.927	1	0.927	drs	11	1	1	1	-	1	↓
Fujian	0.873	0.875	0.999	irs	15	0.804	0.847	0.949	irs	17	↑
Jiangxi	0.956	0.98	0.976	drs	10	0.987	0.995	0.993	drs	8	↓
Shandong	0.82	1	0.82	drs	19	0.87	1	0.87	drs	12	↓
Henan	0.835	0.947	0.882	drs	17	0.839	0.939	0.893	drs	14	↓
Hubei	0.917	0.991	0.925	drs	12	0.941	0.98	0.96	drs	9	↓
Hunan	0.826	0.882	0.936	drs	18	0.858	0.892	0.963	drs	13	↓
Guangdong	1	1	1	-	4	1	1	1	-	1	-
Guangxi	0.981	1	0.981	drs	9	1	1	1	-	1	↓
Hainan	0.694	0.864	0.804	irs	23	0.54	0.987	0.547	irs	25	↑
Chongqing	1	1	1	-	5	1	1	1	-	1	-
Sichuan	0.992	1	0.992	drs	8	1	1	1	-	1	↓
Guizhou	1	1	1	-	6	1	1	1	-	1	-
Yunnan	1	1	1	-	7	0.927	0.992	0.935	irs	10	↑
Tibet	0.373	1	0.373	irs	31	0.102	0.507	0.201	irs	31	↑
Shanxi	0.595	0.614	0.969	irs	26	0.563	0.624	0.902	irs	23	↑
Gansu	0.761	0.767	0.993	irs	21	0.691	0.789	0.875	irs	19	↑
Qinghai	0.707	1	0.707	irs	22	0.523	1	0.523	irs	26	↑
Ningxia	0.776	1	0.776	irs	20	0.464	1	0.464	irs	29	↑
Xinjiang	0.648	0.714	0.908	irs	24	0.58	0.703	0.826	irs	22	↑
mean	0.802	0.876	0.92			0.747	0.871	0.847			

drs indicates diminishing returns to scale, irs indicates increasing returns to scale, and - indicates constant returns to scale.

#### 5.1.2 Stage 2: SFA regression analyses

The effect of environmental variables on the input slack variables was measured using the input slack variables calculated in the first stage of the DEA analysis as the dependent variables. The independent variables were GDP per capita, population density, urbanization rate, and government health expenditures. In the second stage of SFA regression analysis, the positive and negative regression coefficients are inversely proportional to the increase or decrease in efficiency. If the regression coefficient is positive, it means that an increase in that independent variable will bring about an increase in the slack variable, which will lead to wasted resources and decreased efficiency. If the regression coefficient is negative, an increase in the independent variable will lead to a decline in the slack variable, which will lead to reduced waste of resources or increased output, which will, in turn, improve efficiency. Frontier 4.1 software was used to perform the calculations, and the analysis results are shown in Table 4.

As shown in Table 5, the level of GDP per capita and population density have a positive impact on efficiency gains, while urbanization and government health expenditures can hurt efficiency gains. Rising economic levels and increasing population density are conducive to growth in the efficiency of primary healthcare institutions. Regions with a higher level of economic development have relatively high government health expenditures, adequate input of PHC resources, high population densities, and a corresponding growth in demand for basic medical and healthcare services, thus facilitating the use of health resources.

**Table 5:** SFA regression results.

	**Number of health institutions**	**Number of health personnel**	**Number of beds**
Cons	−1.0162E+04^***^	−6.0525E+03^***^	−1.8567E+04^***^
GDP per capital	−5.1673E+04^***^	−3.7207E+04^***^	−1.0489E+05^***^
Population density	−4.4394E+03^***^	−6.8401E+03^***^	−1.8422E+04^***^
Urbanization rate	2.4953E+04^***^	2.1162E+04^***^	6.2203E+04^***^
Government health expenditure	1.0416E+04^***^	3.5205E+02^***^	5.5747E+03^***^
σ^2^	2.2896E+08^***^	8.4459E+07^***^	5.6580E+08^***^
γ	1.00	1.00	1.00
LR test	1.4197E+01	1.4245E+01	1.4197E+01

^***^ indicate significance at the 1% levels, respectively.

In terms of negative impacts, the process of urbanization in China has brought about huge population movements, with a large number of laborers moving to the cities, leading to an increase in the demand for medical services in community health service centers for urban residents, while the decrease in the resident population in rural areas has led to a decrease in the demand for medical services in township health hospitals and village health clinics, which has led to a decrease in efficiency. Although the Chinese government's expenditures on the PHC system increased from 2012 to 2020 to compensate for its gaps in funding, equipment, and personnel (34, 69), owing to the wide disparities in regional development, sustained inputs may also lead to resource redundancies and require differentiated treatment for some regions.

#### 5.1.3 Stage 3: DEA analysis again

**Figure 2:**
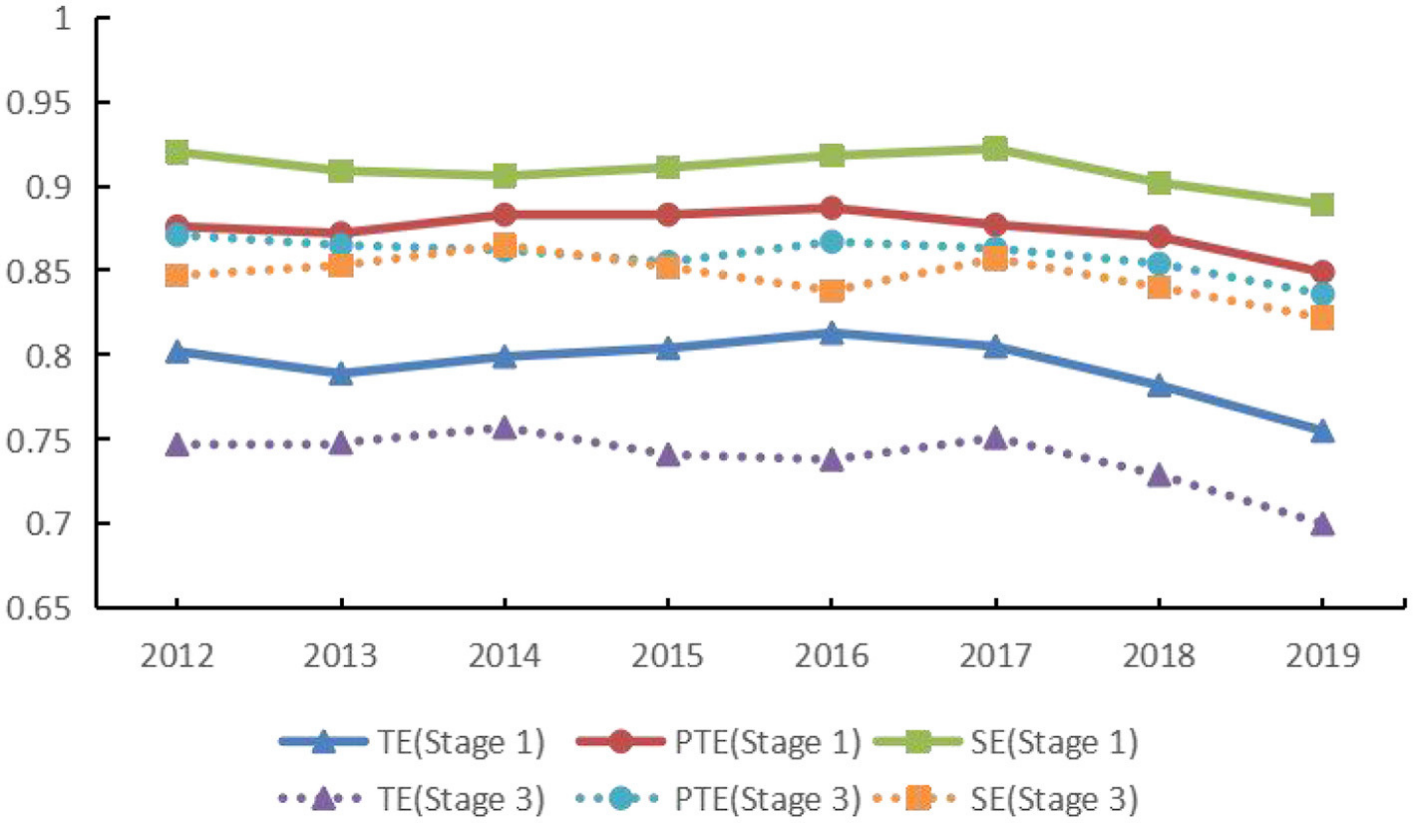
Comparison between stage 1 and stage 3 efficiencies (2012–2020).

After employing Stochastic Frontier Analysis (SFA) regression to treat the input–output variables, the DEA analyses were reconducted. The results demonstrate a notable decline in the SFA-adjusted DEA outcomes compared with those from the initial traditional DEA analysis (Figure 2). Specifically, the mean value of comprehensive efficiency decreased from 0.787 to 0.732, pure technical efficiency from 0.871 to 0.857, and scale efficiency from 0.906 to 0.841. These findings suggest that traditional DEA analysis might overestimate the efficiency levels of the primary healthcare system by failing to account for random disturbances and environmental factors. Moreover, these results indicate that with shifts in the socioeconomic landscape, enhancing the efficiency of China's primary healthcare services presents increasing challenges and warrants greater focus.

#### 5.1.4 Interprovincial heterogeneity

A comparative analysis of the efficiency of primary healthcare service systems across 31 provincial units in mainland China highlights significant disparities in the mean efficiency values among provinces. For example, in the third stage, provincial units in Zhejiang, Anhui, Guangdong, and Guangxi reached DEA efficiency (comprehensive efficiency value = 1), while Tibet's complete efficiency was only 0.102 (Table 3). The gap in efficiency values between provinces is too wide for coordinated regional development, and there is a need to take measures to narrow the gap by focusing on support to less efficient provinces through policy interventions.

In terms of returns to scale, provincial units in a state of increasing returns to scale still account for the vast majority of provinces, indicating that most provinces can still improve their efficiency by investing more in PHC resources. In formulating specific resource allocation policies, it is crucial to consider the dynamic challenges faced by different provinces to avoid redundancy and waste of resources.

**Figure 3:**
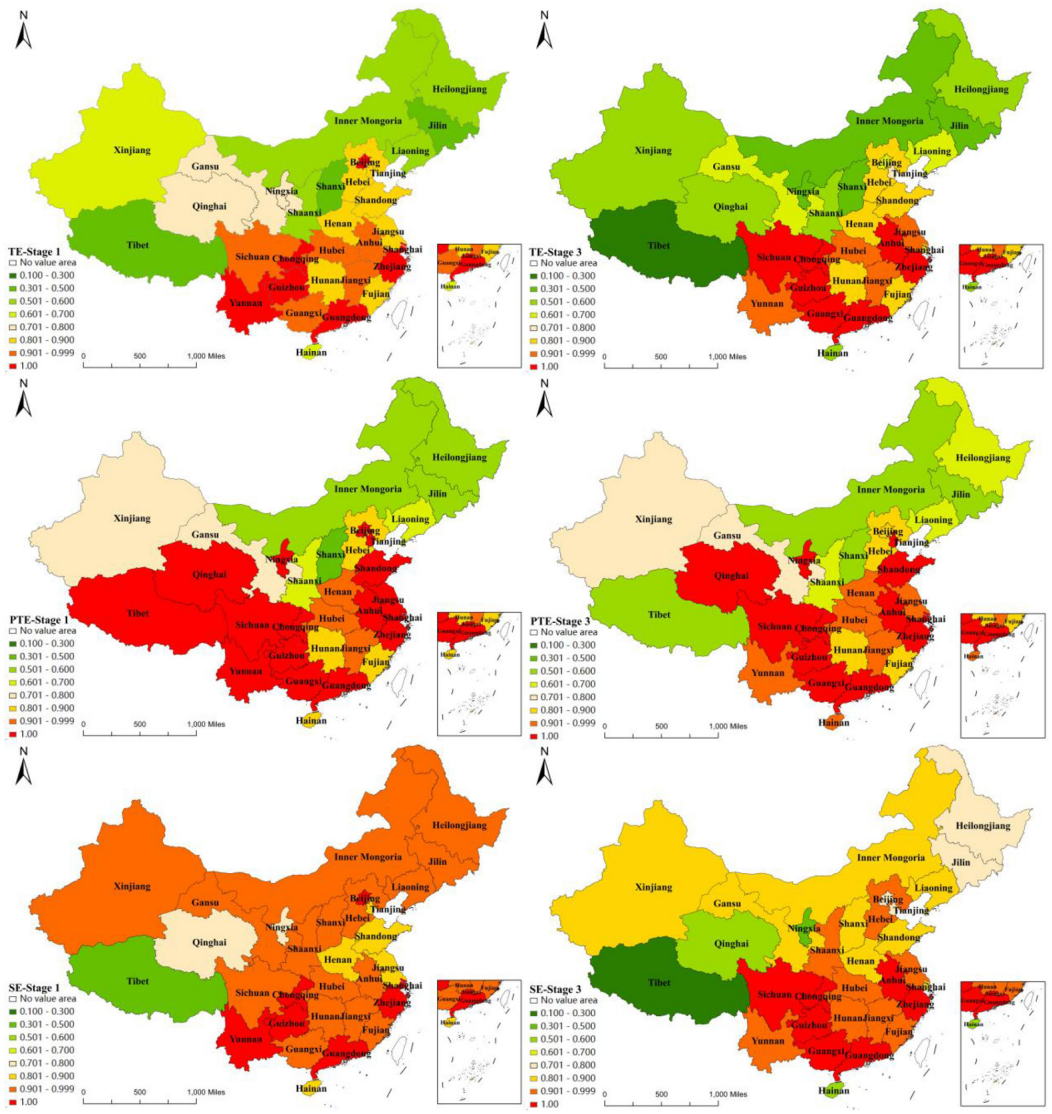
Comparison between stage-1 and stage-3 efficiency in 31 provincial units.

The results in Figure 3 show the regional disparities in PHC services in 31 provinces and cities in mainland China. We can find that the mean efficiency of the provinces in the east-central and southwestern regions of the Yangtze River is much higher than that of the areas to its north. The better efficiency profiles are mostly found in the East Midlands. The Chinese mainland is a vast region with large disparities in regional economic development, with the level of economic and social development in the east and central regions higher than that in the west. This also reflects the impact of factors such as economic and social development on the efficiency of PHC services.

### 5.2 Dynamic efficiency analysis

To further understand the dynamics of efficiency, the third stage of adjusted efficiency results was dynamically analyzed using the Malmquist index. The study found that from 2012 to 2020, the average value of the total factor productivity change index (TFPCH) for China's PHC system was 0.955 or an average annual decline of 4.5%. The mean value of the combined technical efficiency change index (EFFCH) was 0.983, i.e., an average annual decline of 1.7%. The mean value of the pure technical efficiency change index (PECH) was 0.994, i.e., an average annual decrease of 0.6%, and the mean value of the scale efficiency change index (SECH) was 0.989, i.e., an average annual decrease of 1.1%. The average value of the technological progress change index (TECHCH) was 0.971, with an average annual decline of 2.9%, which indicates that technological progress somewhat constrains the improvement of total factor productivity and that efficiency can be further enhanced by improving technological innovation capacity, management level, and resource utilization (Table 6). In this process, enhancing investment in manpower training and technological research and development is essential. High-quality human resources and the deployment of advanced technologies can directly improve the efficiency and quality of services.

**Table 6:** Dynamics of efficiency of PHC services (2012–2020).

**Year**	**EFFCH**	**TECHCH**	**PECH**	**SECH**	**TFPCH**
2012–2013	1	1.031	0.992	1.007	1.031
2013–2014	1.017	0.974	0.994	1.023	0.991
2014–2015	0.971	0.953	0.992	0.978	0.926
2015–2016	0.996	1.007	1.017	0.979	1.003
2016–2017	1.027	1	0.996	1.032	1.027
2017–2018	0.96	0.965	0.989	0.97	0.926
2018–2019	0.947	1.015	0.975	0.971	0.961
2019–2020	0.954	0.837	0.999	0.955	0.798
Mean	0.983	0.971	0.994	0.989	0.955

### 5.3 Spatial autocorrelation analysis

#### 5.3.1 Global autocorrelation

**Figure 4:**
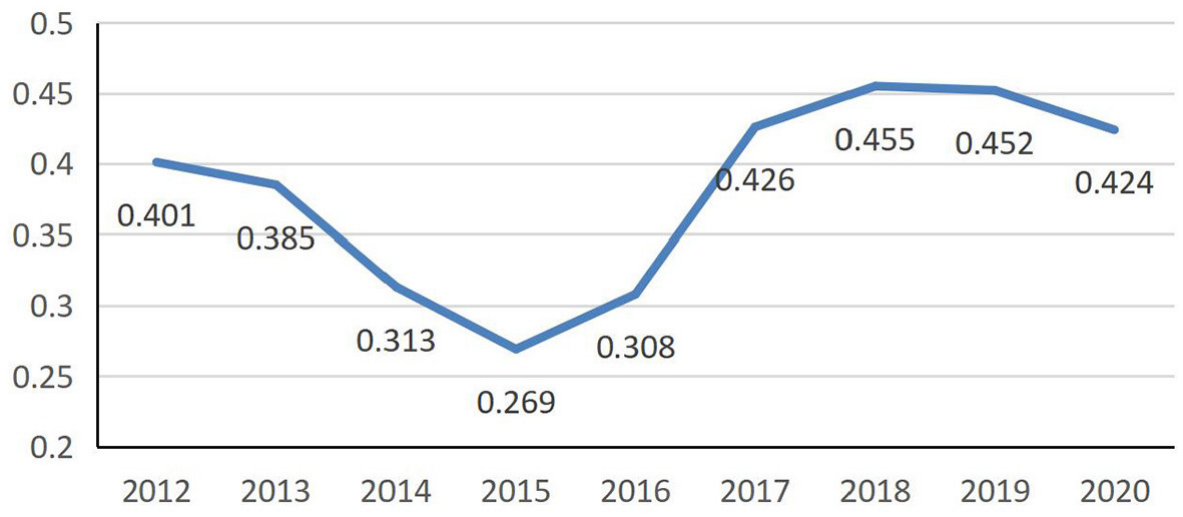
Global Moran's I index for the PHC system (2012–2020).

Spatial correlations in PHC system efficiency in 31 provincial units from 2012 to 2020 was studied using Moran's I index. The three-stage DEA model was adjusted based on the efficiency values, and the neighbor distance matrix measured it. The results showed that Moran's I index was positive from 2012 to 2020, indicating a positive spatial correlation of PHC efficiency in the 31 provincial units (Table 5). From 2012 to 2020, Moran's I index decreased from 0.401 to 0.424 (Table 7), showing a floating upward trend, i.e., spatial correlation increased slightly, and spatial heterogeneity decreased somewhat. Specifically, the global Moran's index values were distributed in a U-shaped structure before COVID-19 and declined after the outbreak (Figure 4), reflecting the impact of COVID-19 on the spatial correlation of efficiency. This highlights the impact of external shocks on the spatial relationships of efficiency, which may distort the understanding of spatial dynamic efficiency over time.

**Table 7:** Global Moran's I index of efficiency of the PHC system.

**Year**	**Moran's I**	**p_value**	**Year**	**Moran's I**	***p*_value**
2012	0.401	0.000	2017	0.426	0.000
2013	0.385	0.000	2018	0.455	0.000
2014	0.313	0.000	2019	0.452	0.000
2015	0.269	0.001	2020	0.424	0.000
2016	0.308	0.032			

The spatial correlation decreased yearly after 2012, reaching its lowest value (0.269) in 2015, and it then increased annually, reaching its highest value (0.455) in 2018. This suggests that further research is needed to examine whether regional cooperation and resource-sharing mechanisms contribute to the observed spatial clustering of efficiencies. Analyzing these dynamics could provide policymakers with a deeper understanding of the benefits of interregional cooperation, enabling them to refine their strategies to optimize resource allocation and improve overall health service efficiency.

#### 5.3.2 Local autocorrelation

**Figure 5:**
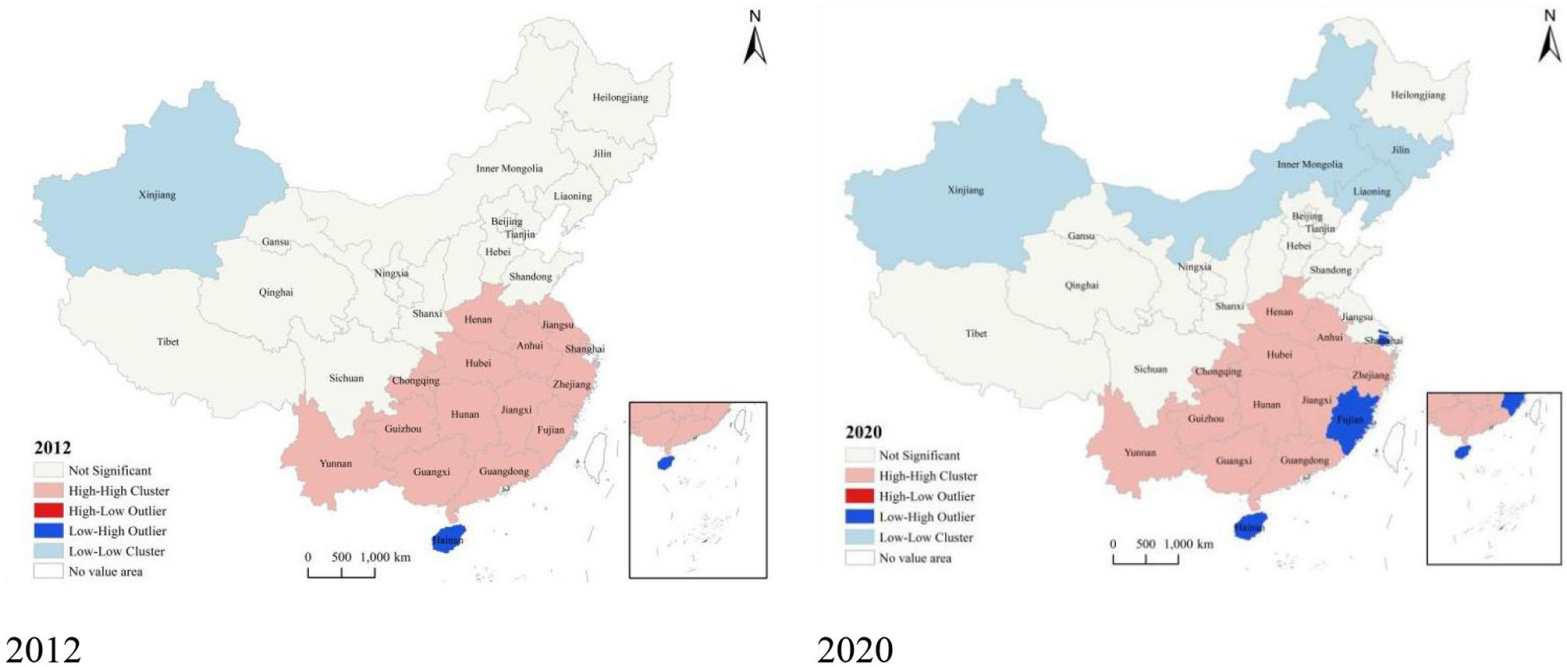
LISA map of PHC system efficiency.

To further study the spatial pattern of the efficiency distribution among the regional units, we continued to calculate the local Moran's I index value. As shown in Figure 5, the units' aggregation mostly remained the same from 2012 to 2020. Specifically, in 2020, the units in a high–high agglomeration included Henan, Anhui, Hubei, Chongqing, Zhejiang, Jiangxi, Hunan, Guizhou, Yunnan, Guangxi, and Guangdong, most of which are concentrated in the south of the Yangtze River. These units were better situated regarding PHC service efficiency and had a solid positive radiation-driven effect on neighboring provinces. However, the over-concentration of resources in high-efficiency areas may exacerbate service shortages and inefficiencies in other regions, hindering the balanced development of the healthcare system. Consequently, formulating policies to equitably distribute resources, along with bolstering support and investment in low-efficiency areas, is crucial for enhancing the overall efficiency and equity of the nation's healthcare services.

In 2012, only Xinjiang was in the low–low agglomeration. While by 2020, in addition to Xinjiang, Inner Mongolia, Jilin, and Liaoning are also fall into low–low agglomeration areas. This trend highlights an intensification of the Matthew effect, where resources and efficiencies increasingly cluster in more developed regions, exacerbating the marginalization of less developed areas. The inefficiency of primary healthcare services in these regions may have worsened interregional imbalances, underscoring that support and resource investment in these areas are insufficient. This situation demands the attention of policymakers and necessitates the implementation of measures to prevent further widening of regional development disparities.

### 5.4 Sensitivity analysis and model validation

To comprehensively assess the reliability of the three-stage DEA model used in this study, robustness tests for internal and external validity were conducted. Internal validity was evaluated through sensitivity analysis to determine the model's responsiveness to the omission of key variables (70). External validity was examined by analyzing the stability of the model over different time points, specifically through the variance in the distribution of efficiency scores across years (70).

In evaluating internal validity, we systematically excluded certain variables from the model, such as the number of institutions, beds, staff, urbanization rate, and government health expenditure. This approach allowed us to assess the impact of these key input variables on the mean efficiency scores (57). The results of the Wilcoxon signed-rank test indicated that while the removal of certain variables (Table 8), such as the number of health staff, led to minor changes in efficiency scores, these changes were not statistically significant (*p-*value = 0.974). Furthermore, both Cohen's d-value and Spearman rank correlation coefficients suggested that the exclusion of each variable had a minimal effect on the overall efficiency score of the model. This consistency underscores the model's resilience to changes in input variables, reinforcing the validity of its internal structure.

**Table 8:** Robustness test of the model.

**Panel A: sensitivity analysis of the Three-stage DEA model**				
**Variable/removed**	**Average scores**	***p*** **value(Wilcoxon)**	**Cohen's d**	**Spearman rank correlation(sig.)**
None	0.747	-	-	-
Number of health institutions	0.735	0.103	0.194	0.887 (0.000)
Number of beds	0.708	0.804	0.071	0.880 (0.000)
Number of health personnel	0.697	0.974	0.021	0.842 (0.000)
Urbanization rate	0.726	0.465	0.204	0.838 (0.000)
Government health expenditure	0.738	0.156	0.142	0.827 (0.000)
**Panel B: the distribution variance of efficiency scores**				
**Year**	***p*** **-value(Wilcoxon)**	**Cohen's d**	**Spearman rank correlation (sig.)**	
(2012-2013)	0.809	0.003	0.936(0.000)	
(2013-2014)	0.602	0.018	0.951(0.000)	
(2014-2015)	0.388	0.065	0.927(0.000)	
(2015-2016)	0.57	0.012	0.985(0.000)	
(2016-2017)	0.43	0.051	0.984(0.000)	
(2017-2018)	0.253	0.088	0.978(0.000)	
(2018-2019)	0.102	0.114	0.974(0.000)	
(2019-2020)	0.294	0.072	0.980(0.000)	

External validity was assessed by analyzing the distribution of efficiency scores between consecutive years using ANOVA (71). Changes in efficiency scores from 2012 to 2020 were evaluated with the Wilcoxon signed-rank test, which revealed no statistically significant differences in the distribution of efficiency scores between any consecutive years. Cohen's d-values ranged from 0.003 to 0.114, and the significance of the Spearman rank correlation coefficients (*p* < 0.001) further confirmed the stability and external consistency of the model's scores. These results indicate that despite potential policy changes or fluctuations in the external environment during the study period, the model demonstrated high stability in relation to annual variations, thereby enhancing its external validity.

## 6 Discussion

Using panel data from 31 provincial units in mainland China spanning from 2012 to 2020, this study developed an index system to evaluate the efficiency of the primary healthcare service system and conducted an empirical analysis using a three-stage DEA-Malmquist model. The study yielded several key findings: first, the efficiency of primary healthcare services in China remains suboptimal and has not shown significant improvements over time, aligning with numerous previous studies (8, 69). While earlier research generally indicated that scale efficiency was relatively robust, and that pure technical efficiency was the primary constraint (34, 72), this study challenges these conclusions. By applying SFA regression to eliminate the effects of random disturbances and environmental factors, it was found that traditional DEA methods tend to overestimate scale efficiency. Both scale and pure technical efficiencies were identified as significant constraints on overall efficiency, suggesting that both areas require further optimization and enhancement to significantly improve the comprehensive efficiency of primary healthcare services in China.

Second, this study revealed that GDP per capita and population density positively influenced the efficiency of the primary healthcare delivery system, while urbanization levels and government health expenditures appeared to have detrimental effects. The positive impact of GDP per capita and population density on primary healthcare efficiency corroborates the findings from the study by Zhou et al. (31). Conversely, research study by Zhong et al. in Hunan province indicated that changes in GDP per capita did not enhance efficiency, while investigation by Tian et al. in Hainan province found an inverse relationship between population density and technical efficiency (73). These findings underscore the necessity for tailored planning that accounts for the unique conditions of different regions.

Regarding the negative impacts of urbanization levels, this is consistent with the study by Zhang et al., which showed that the higher the level of urbanization is, the more input redundancy is generated (74). China's urbanization has been accompanied by great labor force mobility, mainly rural to urban (75). The supply of basic medical facilities usually lags behind the growth rate of the population, and this, together with the problem of uneven distribution of public services between urban and rural areas, exacerbates the mismatch between the basic medical needs of urban and rural residents and the supply of primary medical care facilities.

In addition, it is worth noting that the most previous studies have concluded that government health expenditure positively affects healthcare efficiency (18), but our findings yield the opposite conclusion. This may be for the following two reasons: First, there are pronounced regional differences in Chinese government health expenditure (76), with an overall tendency to favor regions with higher economic levels (77), and given regional disparities in economic development, government health expenditure has had other impacts on PHC institutions of various regions. Second, COVID-19 has posed a significant challenge to the PHC system (10, 78), and efficiency must be improved despite the continuous increase in government investment in health. Of course, this situation may change after COVID-19.

Then, this study underscores a substantial disparity in the efficiency of primary healthcare services across different regions of China, accompanied by a positive spatial correlation. The efficiency levels among the 31 provincial units vary widely, and given China's extensive geographic expanse, these variances manifest as pronounced regional differences (79). Upon categorizing the 31 provinces into three regions, namely, eastern, central, and western and comparing the rankings of overall efficiency, it was observed that the order of efficiency sequence is as follows: eastern > central > western (Table 9). This finding aligns with the research conducted by Li et al. (79) and further highlights the uneven distribution and utilization of healthcare resources across the country (80).

**Table 9:** Comparison of resource efficiency of PHC system in East, Central, and West China.

**Region**	**Before**			**After**		
	**TE**	**PTE**	**SE**	**TE**	**PTE**	**SE**
East	0.873	0.927	0.943	0.796↓	0.926↓	0.863↓
Central	0.738	0.791	0.936	0.760↑	0.825↑	0.906↓
West	0.779	0.886	0.888	0.694↓	0.851↓	0.794↓

Finally, our study reveals spatial correlations in the efficiency distribution of China's interprovincial primary healthcare delivery system. Most provincial units within the high–high agglomeration area are situated in the east-central region south of the Yangtze River, particularly in the central region where a higher proportion of provincial units is found. In contrast, provinces in the low–low agglomeration area are primarily located in the northwestern and northeastern regions. Research by Guo et al. corroborates this, indicating that the east-central region benefits from relatively better efficiency in health resource allocation, which favorably supports the development of its primary healthcare service system. However, the inefficiencies observed in the northwest and northeast should draw policymakers' attention. Augmenting public health resources in isolation may not suffice to address inefficiencies effectively; instead, thorough studies into varying regional management practices and healthcare needs are essential to ensure more effective resource utilization. Given the economic and geographical constraints, the development of primary healthcare service systems in these regions could be enhanced through infrastructure improvements (81) and the advancement of telemedicine (80, 82).

## 7 Conclusion

This study conducted a comprehensive empirical analysis of the efficiency of China's primary healthcare service system using data from the official statistical yearbook for 31 provincial units in mainland China from 2012 to 2020, employing a three-stage DEA-Malmquist model. Additionally, the spatial patterns of efficiency were analyzed using the Moran index. The key findings are as follows:

1. From 2012 to 2020, the efficiency level of China's primary healthcare service system declined, indicating that both pure technical efficiency and scale efficiency require enhancement.

2. In terms of regional distribution, the efficiency of primary healthcare services in mainland China ranks as follows: east > central > west. Efficiency in more developed regions significantly surpasses that in less developed ones.

3. The distribution of efficiency exhibits a spatial correlation, where regions with high efficiency demonstrate a strong radiative influence, whereas regions with low efficiency have a limited impact, suggesting a Matthew effect.

4. The level of GDP per capita and population density positively influences efficiency increases, whereas urbanization levels and government health expenditures negatively impact efficiency.

The study utilized a three-stage DEA analysis and observed a decrease in efficiency after accounting for external disturbances and environmental factors. This not only extends the application of the methodology but also highlights the significant impact of external disturbances on efficiency. Moreover, the spatial analysis of efficiency provides additional insights for fostering coordinated development across primary healthcare services. These findings offer a scientific foundation and reference for China and other nations striving to enhance the efficiency of their primary healthcare services. Our study also has some limitations. The first concerns the construction of the input and output indicator system. The scope of services of PHC organizations in China includes medical and healthcare services and public healthcare services. Nevertheless, since data on the indicators of public healthcare services at the provincial level are not available, only the indicators of medical and healthcare services were included in this study for measurement. Second, regarding the influential factor of population density, only the resident population was considered, not the floating population.

Future research should focus on synthesizing indicators and the subjects of study to enhance the construction of the indicator system. Additionally, the trends observed in the regional distribution of this study suggest that internal regional policy differences and the allocation of health resources may significantly influence efficiency. Consequently, future investigations should delve into the effects of potential factors beyond economic development levels on efficiency. This expanded focus could provide a more comprehensive understanding of the variables affecting healthcare efficiency and inform more targeted and effective policy interventions.

## Data availability statement

The original contributions presented in the study are included in the article/supplementary material, further inquiries can be directed to the corresponding author.

## Author contributions

RH: Writing – original draft, Conceptualization. WL: Writing – review & editing, Writing – original draft. BS: Writing – original draft, Data curation, Methodology. HS: Writing – original draft, Data curation, Methodology. JH: Writing – review & editing, Data curation, Methodology. CZ: Writing – review & editing, Validation. JC: Writing – review & editing, Validation. All authors contributed to the article and approved the submitted version.
